# Prevalence of BRCA mutation in breast and ovarian cancer among women in India: A systematic review and meta-analysis protocol

**DOI:** 10.1371/journal.pone.0306612

**Published:** 2024-07-16

**Authors:** Abhilash Patra, Syeda Sana Ali, Ng Marina Devi, Amatullah Sana Qadeer, Sureshkumar Kamalakannan, Shona Nag, Shriniwas Subhash Kulkarni, Senthil Rajappa, Nisha Hariharan, Hira Ballabh Pant, Varun Agiwal, Nirupama A. Y.

**Affiliations:** 1 E2E Pfizer Fellow, Indian Institute of Public Health, Hyderabad, Telangana, India; 2 Indian Institute of Public Health, Hyderabad, Telangana, India; 3 Department of Social Work, Education and Community Wellbeing, Northumbria University, London, United Kingdom; 4 Oncology Department, Sahyadri Group of Hospitals, Pune, Maharashtra, India; 5 Basavatarakam Indo American Cancer Hospital and Research Institute, Hyderabad, India; CNR, ITALY

## Abstract

**Purpose:**

We present a methodically devised protocol for conducting a systematic review and meta-analysis aimed at ascertaining the prevalence of BReast CAncer gene (BRCA) mutations in breast and ovarian cancer (BOC) among women in India. The review will include cross-sectional, cohort, case-series, and registry-based studies focusing on females clinically diagnosed with any stage of BOC, tested for BRCA germline mutation and undergone any form of treatment.

**Methods:**

A Cochrane literature search will be carried out to identify all the published and unpublished articles available in English from 2010 till date across various electronic databases including PubMed, Psych Info, SCI, Cochrane Central, Embase, Scopus, IND Med and Google Scholar. A step-by-step process will be followed to select all the relevant studies for final inclusion using Rayyan software. The selection process of the review will be reported based on Preferred Reporting Items for Systematic Review and Meta-analysis Protocols (PRISMA) checklist. The protocol has been registered in PROSPERO (ID: CRD42023463452). Joanna Briggs Institute Critical Appraisal Checklist will be used to evaluate the methodological quality of the included studies. The outcome measure will be the prevalence of BRCA1/2 gene mutation in this population. Meta-analysis will be performed to report the pooled prevalence along with 95% confidence interval.

**Discussion:**

The results of this review study will provide valuable insights for clinicians, and policy makers, enabling them to formulate guidelines that underscore the importance of screening for BRCA mutations in cases of BOC.

## Introduction

According to the GLOBOCAN 2020 report, globally, 24.5% of all cancers that occur in women were breast cancer, and ovarian cancer (OC) affects 3.4% of women. The corresponding mortality rates were reported as 15.5% for BC and 4.7% for OC [1]. Notably, in India, the incidence of breast and ovarian cancer (BOC) among females exceeds the global estimates and was reported to be 26.3% and 6.7% respectively; making them the top five most frequent cancers in India [2]. Furthermore, it is estimated that over one-fifth of OC patients and 10% of BC patients worldwide have a suspected hereditary germline mutation [3, 4]. Furthermore, a twofold rise in the occurrence of BOC was noted in the presence of family history among first-degree relatives [5]. The BReast CAncer genes (BRCA) are recognized as the most commonly linked germline mutation [6, 7]. Also, the presence of BRCA1 and BRCA2 gene mutations can lead to an increase in the incidence of BOC, raising the risk of BC by 40–85% and OC by up to 54% [8].

A population-based review study reports that BRCA gene mutation varies widely between populations, that is according to race and ethnicity [9]. However, according to European Society for Medical Oncology (ESMO) clinical practice guidelines reports that the frequency of BRCA1 and 2 mutations among unselected BOC patients for family history or age is less than 1–7% for BRCA1 and 1–3% for BRCA2 [10]. Furthermore, due to epidemiological transition, BC incidence is increasing among young women, that is those under the age of fifty [11].

A substantial cohort study involving Chinese women with BOC, it was reported that 48.6% exhibited deleterious mutations, particularly those with a significant family history of BOC. Also, more than one-fourth of the women had BRCA 1 and BRCA 2 mutations. However, this study reports a higher prevalence of BRCA mutations as it involves high-risk patients and does not reflect the relative frequency of BRCA mutation among Chinese women [12]. A systematic review on the prevalence of BRCA pathogenic variants in BOC in North African women reported more than 50% of pathogenic variants in BOC women, with novel pathogenic variants found to be 23.5% [13].

In India, the most common form of cancer is BOC, which stands as the foremost cause of cancer-related deaths in the country [14]. There is a paucity of data on the prevalence of germline mutation in BRCA1 and BRCA2 genes in BOC in India [15]. There is no aggregated data on the distribution of BRCA 1/2 gene mutation in BOC in India. Moreover, this evidence has the potential to play a pivotal role in shaping a comprehensive global agenda for germline testing and establishing treatment guidelines for hereditary breast and ovarian cancer (HBOC). Additionally, there are different multi-gene sequencing panel tests that highly depend on the different online databases and published literature [15]. Although the importance of germline testing is known among oncologists, the application of the same varies based on the availability of resources. The assessment of prevalence will help in highlighting personalized treatment, early detection, and prevention. It will also help to address the burden of BOC by framing a case for the development of a nationwide policy for germline testing. Hence, the present study aims to quantify the prevalence of BRCA gene mutation in BOC among women in India by consolidating all the available literature to perform a systematic review and meta-analysis.

## Objective

To estimate the prevalence of BRCA mutation in breast and ovarian cancer (BOC) among women in India.

## Methods

This systematic review protocol has been registered in The International Prospective Register of Systematic Reviews (PROSPERO) (ID: CRD42023463452) prior to preliminary search and data extraction.

### Criteria for considering studies for the review

#### Types of studies

Studies that report the prevalence and frequency of BRCA1/2 among women in India will be included. Studies such as cross-sectional, cohort, case-series, and registry-based studies that have the possibility of giving information on the prevalence of BRCA mutation among BOC women will be included. Interventional studies and studies those are not available in the English language will be excluded.

#### Types of participants

Studies in which the female participants have been clinically diagnosed with BOC; are in any stage of disease; have undergone any form of treatment; underwent BRCA germline testing will be included irrespective of age. Male BC patients and studies in which female participants are BRCA positive but have cancers other than BOC will not be considered. Studies on carriers of BRCA mutation will also be excluded.

#### Outcome measures

Prevalence and frequency of BRCA1 or BRCA 2 gene mutation among women diagnosed with BOC, expressed in rates or proportions. Studies that report the prevalence of BRCA mutation through any sequencing panel, as reported by each study author will be included. Prevalence reported without a defined target population (Denominator) will not be included.

### Search strategy

A literature search following Cochrane methodology will be carried out to identify all the published and unpublished articles available in English. We will adhere to the recommendations provided by the oncologists, who are the co-authors of this review, in establishing the timeline from the year 2010 till the date of search. The electronic databases that will be included in the search are PubMed, Psych Info, SCI, Cochrane Central, Embase, Scopus, IND MED and Google Scholar. The concepts “population” and “outcome” were used to develop the search strategy. For the concept “population”, the search terms used were “breast cancer”, “breast neoplasms”, “breast tumour”, “breast carcinoma”, “cancer of breast”, “ovarian cancer”, “ovary neoplasms”, “ovarian neoplasm” “cancer of ovary”, “metastasis”, “malignant”, benign”, “mutation”, “genes”, “BRCA”, “BRCA1, “BRCA2” and “India”. For the concept “outcome”, the search terms used were “incidence”, “prevalence”, “epidemiology”, “survival”, “mortality”, “outcome” and “prognosis”. Boolean operators “AND” and “OR” were used along with truncation to refine the search (S1 File). The reference list of recognized studies will be further explored to search for additional papers. Since the IND MED database is not accessible online, the search process will involve identifying the Indian journals listed in IND MED and conducting hand searches within each journal. Furthermore, an extensive search of grey literature will be conducted in accordance with the specified timelines.

### Data collection and analysis

Rayyan software will be utilized to systematically choose all the pertinent studies for inclusion through a methodical selection process.

#### Selection of studies

Firstly, all the studies for inclusion in the review will be screened and duplicates will be removed. All four reviewers AP, SA, MD, SQ will independently screen the title of the studies. Those that fit the inclusion criteria will be considered for the next stage of screening and the rest will be excluded. AP, SA, MD and SQ will independently screen the abstract of the selected studies. All the eligible studies full texts will be retrieved, and all reviewers will independently assess the full papers for their eligibility for final inclusion and data extraction. If a disagreement arises during the selection process, the majority of the consensus will be considered for inclusion of the study in the review. The selection process of the studies will be depicted in the flow diagram following the Preferred Reporting Items for Systematic Reviews and Meta-Analyses (PRISMA) guidelines.

#### Data extraction

A proforma for extracting data from the included studies will be developed and pre-tested. AP and SA will independently extract data from the selected full-text article. This will be cross-checked by MD and SQ. If there is any disagreement, an expert’s opinion will be drawn from NAY. The data extracted will be collected in MS Excel.

#### Methodological quality assessment

The methodological quality of the included studies will be assessed using the Joanna Briggs Institute Critical Appraisal Checklist [16] This tool comprises nine items, covering aspects such as sampling frame, technique, sample size, subjects, settings, and data collection tools for assessment. Two reviewers NG and SA will independently assess the data based on the checklist items, and this assessment will be verified by AP and SQ. In cases where needed, expert consensus will be sought from a fifth reviewer (NAY).

### Data analysis

The meta-analysis will be conducted using Stata version 14, employing the "metaprop" command. This will allow for the calculation of the combined prevalence of BRCA mutations along with its corresponding confidence interval. To gauge heterogeneity, Q statistics, degrees of freedom (df), and I2 statistic will be employed. Furthermore, a funnel plot and Egger’s test will be utilized to evaluate potential publication bias.

### Data synthesis & meta-analysis

The analysis will be conducted with assumption of a random-effects model, providing a more comprehensive estimate compared to a fixed-effects model. Findings will be elucidated in relation to the pooled prevalence, accompanied by a 95% confidence interval and a visual representation through a forest plot. The forest plots will depict proportions along with their corresponding confidence intervals (CI). Additionally, subgroup analysis will be performed, panel testing, study design, and states within India, as well as based on selected and unselected populations. If we get homogenous studies we will conduct meta-analysis. If not, we will narratively synthesise and present the data.

### Status and timeline of the study

The review is currently in progress and is anticipated to conclude, with results expected to be reported within six months.

## Discussion

This systematic review and meta-analysis will provide robust evidence and valuable insights for formulating and enhancing guidelines to optimize screening approaches for genetic mutations associated with BOC. The outcomes will offer valuable guidance to healthcare professionals, nurses, in comprehending genetic mutations in these types of cancer. Furthermore, this study will underscore the broad significance of genetic testing in healthcare, emphasizing its relevance for treatment planning and preventive measures. It will also underscore the necessity for tailored guidelines and its integration into national health programs.

To ensure the highest quality of evidence, we have adhered strictly to systematic review and meta-analysis guidelines. This is to provide a more accurate prevalence estimate of BRCA mutations among cases of BOC in India. Our data search and extraction process has been refined through an iterative approach with input from oncology experts. We utilized over 20 keywords across various databases including PubMed, Psych Info, SCI, Cochrane Central, Embase, Scopus, IND MED, and Google Scholar. The search results will be meticulously de-duplicated and screened by five reviewers and experts to ensure the data’s comprehensiveness and reliability.

Despite the merits of our research as mentioned above, there are significant challenges in developing and implementation of this review protocol and subsequently the meta-analysis. Given the complex nature of BRCA mutations in both breast and ovarian cancer, establishing inclusion and exclusion criteria presents a notable challenge. Additionally, the process of screening for germline mutations in these cancers is intricate, demanding meticulous attention to ensure the accuracy and representativeness of the evidence supporting our research. The preliminary database search yielded over 1000 research studies with considerable overlap, underscoring the need for caution in selecting data during the extraction process. Incorporating relevant articles not indexed in electronic databases, often referred to as grey literature, also poses a challenge. Furthermore, the scarcity of research on the prevalence of BRCA mutations in BOC in India complicates the integration and analysis of available data.

## Supporting information

S1 File Search strategy. (DOCX)

S1 Checklist PRISMA-P 2015 checklist. * It is strongly recommended that this checklist be read in conjunction with the PRISMA-P Explanation and Elaboration (cite when available) for important clarification on the items. Amendments to a review protocol should be tracked and dated. The copyright for PRISMA-P (including checklist) is held by the PRISMA-P Group and is distributed under a Creative Commons Attribution Licence 4.0.
(DOC)
