# Supplementary material for: Prevalence of BRCA mutation in breast and ovarian cancer among women in India: A systematic review and meta-analysis protocol
Source: PLoS One. 2024 Jul 16;19(7):e0306612. doi: 10.1371/journal.pone.0306612 (PMC11251578; doi:10.1371/journal.pone.0306612)
Supplement: S1 File — (DOCX) [file pone.0306612.s001.docx]

**Supplementary File-1**

**Search Strategy**

**PubMed**

((((((("Mutation"[MeSH Terms])) OR (mutation*[Title/Abstract])) OR (((((((Genes[MeSH Terms]) OR (BRCA1[Title/Abstract])) OR (BRCA2[Title/Abstract])) OR (BRCA1 gene*[Title/Abstract])) OR (BRCA2 gene*[Title/Abstract])) OR (BRCA1 protein[MeSH Terms])) OR (BRCA2 protein[MeSH Terms])))) AND ((((((((((("Breast Neoplasms"[MeSH Terms]) OR (Breast can*[Title/Abstract])) OR (breast tumo*[Title/Abstract])) OR (breast tumo*[Title/Abstract])) OR ((tumo*) AND (breast[Title/Abstract]))) OR (breast carcinoma[Title/Abstract])) OR ((neoplas*) AND (breast[Title/Abstract]))) OR (cancer of the breast[MeSH Terms])) OR (breast neoplasms[Title/Abstract])) OR ((cancer*) AND (breast[Title/Abstract]))) OR (((((((((("Ovarian Neoplasms"[MeSH Terms]) OR (Ovarian can*[Title/Abstract])) OR (Ovarian tumo*[Title/Abstract])) OR (Ovary tumo*[Title/Abstract])) OR ((tumo*) AND (Ovar*[Title/Abstract]))) OR (Ovar* carcinoma[Title/Abstract])) OR ((neoplas*) AND (Ovarian[Title/Abstract]))) OR (Cancer of the Ovary[MeSH Terms])) OR (ovarian neoplasms[Title/Abstract])) OR ((cancer*) AND (ovarian[Title/Abstract])))) AND (India*) AND ((((prevalence) OR (incidence)) OR (frequency)) OR (epidemiology[MeSH Terms])) AND ((data[Filter]) AND (2010:2023[pdat]))
